# Cholesterol, high-density lipoprotein, and glucose index as a cardiometabolic marker associated with heart rate variability and 1-year cardiovascular rehospitalization in chronic coronary syndromes with comorbid anxiety: a retrospective cohort study

**DOI:** 10.3389/fendo.2026.1871491

**Published:** 2026-06-18

**Authors:** Yuan Gao, Yiwei Xu, Chuxin Lyu, Yuhan Ding, Siyuan Yin, Ruijie Shi, Jiemei Zhou, Haowen Zhang, Xiaohu Chen

**Affiliations:** 1 Department of Cardiology, Affiliated Hospital of Nanjing University of Chinese Medicine, Nanjing, China; 2 Department of Cardiology, Jiangsu Province Hospital of Chinese Medicine, Nanjing, China; 3 First Clinical Medical College, Nanjing University of Chinese Medicine, Nanjing, China; 4 School of Integrated Chinese and Western Medicine, Nanjing University of Chinese Medicine, Nanjing, China; 5 Department of Cardiology, Suzhou Hospital, Xiyuan Hospital of China Academy of Chinese Medical Sciences, Suzhou, China; 6 Health Preservation and Rehabilitation College, Nanjing University of Chinese Medicine, Nanjing, China

**Keywords:** anxiety, cardiovascular rehospitalization, cholesterol, high-density lipoprotein, and glucose index, chronic coronary syndromes, heart rate variability

## Abstract

**Background:**

Metabolic dysregulation and autonomic imbalance may be related to adverse outcomes in patients with chronic coronary syndromes (CCS) and comorbid anxiety. We investigated the associations of the cholesterol, high-density lipoprotein, and glucose (CHG) index with a composite heart rate variability measure (HRV_z) and 1-year cardiovascular rehospitalization in this high-risk population.

**Methods:**

This single-center retrospective cohort study included 1020 hospitalized patients with CCS and comorbid anxiety between January 2022 and January 2025. Among them, 571 patients with available 24-h Holter recordings were included in exploratory HRV-related analyses. Demographic, laboratory, Holter-derived HRV, and 1-year cardiovascular rehospitalization data were collected. Missing data were handled using multiple imputation. Multivariable linear and logistic regression models, together with restricted cubic splines, were used to examine linear and nonlinear associations.

**Results:**

Compared with patients in the lowest CHG quartile (Q1), those in the highest quartile (Q4) had significantly lower HRV_z in the Holter subgroup (β = -0.529, 95% CI -0.698 to -0.361; P < 0.001). In the overall cohort, each 1-unit increase in CHG was associated with a higher risk of 1-year cardiovascular rehospitalization (OR = 3.253, 95% CI 2.292–4.617; P < 0.001), and patients in Q4 had a higher risk than those in Q1 (OR = 3.656, 95% CI 2.443–5.472; P < 0.001). In exploratory Holter subgroup analyses, higher HRV_z was associated with lower rehospitalization risk. Restricted cubic spline analyses suggested nonlinear associations among CHG, HRV_z, and rehospitalization risk. Complete-case sensitivity analyses generally supported the main findings, although HRV-related results should be interpreted cautiously.

**Conclusions:**

In patients with CCS and comorbid anxiety, elevated CHG was associated with increased 1-year cardiovascular rehospitalization risk. Exploratory Holter subgroup analyses suggested that higher CHG was associated with lower HRV_z, and lower HRV_z with higher rehospitalization risk. CHG, together with Holter-derived HRV_z, may provide complementary information for risk assessment in this population.

## Introduction

Cardiovascular disease (CVD) remains a leading cause of mortality and disease burden worldwide, with ischemic heart disease accounting for a substantial proportion of this burden. Chronic coronary syndromes (CCS) comprise a spectrum of clinical conditions caused by chronic structural and functional abnormalities of the coronary arteries and/or coronary microcirculation (1). Increasing evidence suggests a close interplay between cardiovascular disease and mental health. Anxiety, depression, and chronic psychological stress are common in patients with cardiovascular disease and are associated with greater symptom burden, poorer treatment adherence, and worse clinical outcomes (2). Previous studies have reported that approximately 20% to 40% of patients with cardiovascular disease experience clinically relevant anxiety symptoms (3–6).

Metabolic abnormalities also play an important role in the progression of coronary artery disease. However, single metabolic markers may not adequately capture the overall burden of glucolipid dysregulation. The cholesterol, high-density lipoprotein, and glucose (CHG) index is a novel composite metabolic marker integrating total cholesterol, fasting blood glucose, and high-density lipoprotein cholesterol. Recent studies have shown that the CHG index is associated with type 2 diabetes, cardiovascular disease, and mortality risk, suggesting its potential value in cardiometabolic risk stratification (7–9). Nevertheless, its clinical significance in patients with CCS and comorbid anxiety remains unclear.

Moreover, autonomic imbalance may be one of the pathways linking psychological stress to adverse cardiovascular outcomes. Heart rate variability (HRV), a classic noninvasive marker of cardiac autonomic regulation, has been widely used to assess sympathovagal balance and prognosis in cardiovascular disease (10). Lower HRV has been observed in patients with anxiety and has also been associated with adverse cardiovascular outcomes (11–13). Compared with single HRV parameters, HRV_z, a composite indicator derived from standardized time-domain HRV measures, may better reflect overall autonomic function.

In patients with CCS and comorbid anxiety, metabolic abnormalities and autonomic dysfunction may be involved in poor prognosis. However, evidence simultaneously evaluating these two dimensions in this specific population remains limited. Therefore, this study aimed to investigate the association between the CHG index and HRV_z, and to further assess the relationships of CHG and HRV_z with 1-year cardiovascular rehospitalization risk in patients with CCS and comorbid anxiety.

## Materials and methods

### Study design and participants

**Figure 1:**
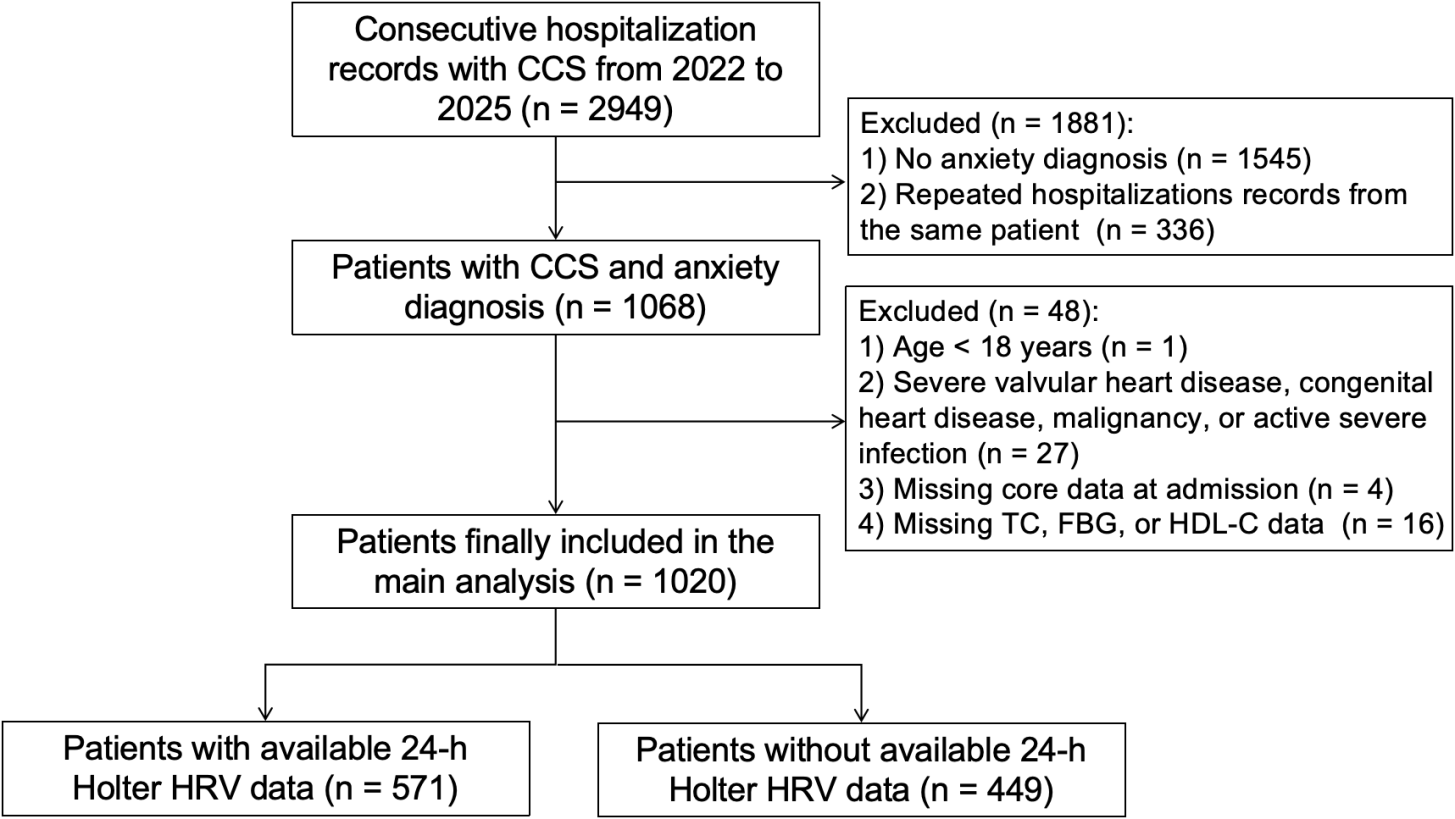
Flowchart of patient selection.

This single-center retrospective cohort study consecutively screened patients who were hospitalized in the Department of Cardiology, Jiangsu Province Hospital of Chinese Medicine, between January 2022 and January 2025 and diagnosed with CCS. Patients with CCS and comorbid anxiety were further included in the present analysis. The inclusion criteria were: (1) age ≥ 18 years; (2) diagnosis of CCS according to the 2024 ESC Guidelines for the management of chronic coronary syndromes; and (3) anxiety was identified based on ICD-11-related discharge diagnoses or final diagnoses recorded in the electronic medical record system during the baseline hospitalization; (4) For patients with multiple hospitalizations, only the first hospitalization record that met the diagnostic criteria was included. Because of the retrospective nature of this study, standardized anxiety scale scores, such as GAD-7 and HAMA scores, as well as information on anxiety severity, specific categories of anxiolytic medications, and treatment adherence, were not available; therefore, further stratified analyses could not be performed. The exclusion criteria were: (1) age < 18 years; (2) severe valvular heart disease, congenital heart disease, malignant tumors, or active severe infection; (3) missing key baseline clinical data; and (4) missing key variables required for calculation of CHG index, including total cholesterol (TC), fasting blood glucose (FBG), or high-density lipoprotein cholesterol (HDL-C); and (5) missing follow-up data. A total of 1020 patients were finally included, of whom 571 had complete 24-h Holter data and were further included in HRV-related analyses (**Figure 1**). The study was conducted in accordance with the Declaration of Helsinki and was approved by the Medical Ethics Committee of Jiangsu Province Hospital of Chinese Medicine (approval No. 2025NL-021-01).

### Data collection

Baseline data were extracted from the electronic medical record system and independently verified by two trained physicians. Demographic variables included age, sex, height, weight, and body mass index (BMI). Clinical history and medication use included hypertension (HTN), type 2 diabetes mellitus (T2DM), cerebral infarction (CI), hyperlipidemia (HLP), chronic kidney disease (CKD), heart failure (HF), atrial fibrillation (AF), and anxiolytic use. Laboratory parameters included TC, FBG, HDL-C, high-sensitivity C-reactive protein (hs-CRP), white blood cell count (WBC), red blood cell count (RBC), hemoglobin (Hb), platelet count (PLT), neutrophil count (NEU), lymphocyte count (Lym), monocyte count (MO), alanine aminotransferase (ALT), aspartate aminotransferase (AST), albumin (ALB), blood urea nitrogen (BUN), creatinine (Cr), uric acid (UA), glycated hemoglobin (HbA1c), triglycerides (TG), low-density lipoprotein cholesterol (LDL-C), apolipoprotein A1 (ApoA1), apolipoprotein B (ApoB), lipoprotein(a) [Lp(a)], high-sensitivity cardiac troponin I (hs-cTnI), N-terminal pro-B-type natriuretic peptide (NT-proBNP), and coagulation-related markers. All laboratory results were obtained from fasting venous blood samples collected on the morning after admission.

Twenty-four-hour ambulatory electrocardiography data were collected in the Holter subgroup. The following time-domain HRV parameters were extracted: standard deviation of all normal-to-normal intervals (SDNN), percentage of adjacent normal-to-normal intervals differing by more than 50 ms (pNN50), root mean square of successive differences between adjacent normal-to-normal intervals (rMSSD), and standard deviation of the average normal-to-normal intervals for each 5-min segment (SDANN).

### Cholesterol, high-density lipoprotein, and glucose index

The CHG index was calculated as follows: CHG = ln{[TC (mg/dL) × FBG (mg/dL)]/[2 × HDL-C (mg/dL)]} (14, 15). Because the original laboratory data were recorded in mmol/L, TC and HDL-C were converted to mg/dL using a factor of 38.67 and FBG using a factor of 18.02 before analysis. Patients were then categorized into quartiles (Q1-Q4) according to the CHG index for baseline comparisons and regression analyses.

### Definition of the composite HRV indicator

To comprehensively reflect overall time-domain HRV, a composite HRV indicator (HRV_z) was constructed using SDNN, SDANN, rMSSD, and pNN50. Because HRV parameters were skewedly distributed, natural logarithmic transformation was applied to SDNN, SDANN, and rMSSD, whereas pNN50 was transformed using log(1 + pNN50) (16–18). The transformed variables were subsequently standardized into Z scores, and HRV_z was calculated as the mean of the four standardized values. Higher HRV_z values indicated higher overall HRV and relatively better autonomic function.

### Outcome

The primary outcome of this study was cardiovascular rehospitalization, defined as readmission to the study center for cardiovascular causes during the 1-year follow-up period after discharge from the baseline hospitalization, with corresponding hospitalization records available in the electronic medical record system of our hospital. Cardiovascular rehospitalization subtypes included acute coronary syndrome, non-acute coronary syndrome ischemic chest pain or suspected myocardial ischemia, worsening heart failure, arrhythmia-related rehospitalization, unplanned revascularization, and other cardiovascular causes. Planned follow-up admissions and hospitalizations for non-cardiovascular causes were not considered primary outcome events.

Because electronic medical records from other hospitals were not available in this retrospective study, rehospitalization events occurring outside the study center were not included in the outcome assessment. Patients without records of the above cardiovascular rehospitalization events in the electronic medical record system of our hospital within 1 year after discharge were classified as not having the primary outcome.

Outcome events were independently reviewed by two investigators based on rehospitalization discharge diagnoses, clinical course records, examination results, and treatment procedures. Any disagreement was resolved by review and confirmation by a third investigator. No independent endpoint adjudication committee was established. Because out-of-hospital death data were not systematically available, patients who did not experience cardiovascular rehospitalization at our hospital during follow-up were treated as not having the primary outcome.

### Statistical analysis

Continuous variables are presented as median with interquartile range (IQR), and categorical variables are presented as number (percentage). Differences across CHG quartiles were compared using the Kruskal–Wallis test for continuous variables and the chi-square test or Fisher’s exact test for categorical variables, as appropriate.

Missing data were handled using multiple imputation by chained equations. In the final analytic models, missing data were limited to BMI and hs-CRP; therefore, only these two covariates were imputed. The CHG components, CHG index, Holter-derived HRV parameters, HRV_z, and 1-year cardiovascular rehospitalization outcome had no missing values and were not imputed. Predictive mean matching was used to impute missing BMI and hs-CRP values. Multiple imputation was performed separately for the overall cohort and the Holter subgroup using the mice package in R. Five imputed datasets were generated with 20 iterations. For HRV-related analyses, HRV_z was constructed from complete Holter-derived HRV parameters before imputation, and a separate imputation procedure was then performed in the Holter subgroup. The imputation model included observed demographic characteristics, clinical history, laboratory parameters, exposure variables, treatment information, Holter availability, and outcome status as predictors, whereas the outcome variable itself was not imputed. Regression estimates from the imputed datasets were pooled using Rubin’s rules. The missing-data pattern, imputation model specification, and representative diagnostic plots are provided in **Supplementary Tables 6, 7** and **Supplementary Figures 1, 2**. Because missingness occurred only in baseline covariates and could be modeled using observed clinical and laboratory information, the missing-at-random assumption was considered reasonable. Complete-case analyses were conducted using listwise deletion according to the variables required for each model and were compared with the main multiple-imputation analyses to assess the robustness of the findings.

In the Holter subgroup, multivariable linear regression was used to evaluate the association between CHG and HRV_z, with HRV_z as the dependent variable. Binary logistic regression was used to assess the association between HRV_z and 1-year cardiovascular rehospitalization. In the full cohort, binary logistic regression was used to examine the association between CHG and 1-year cardiovascular rehospitalization. CHG was modeled both as a continuous variable and as a quartile-based categorical variable. Trend tests were performed by entering CHG quartiles as an ordinal variable. Three models were constructed: Model 1, unadjusted; Model 2, adjusted for age, sex, and BMI; and Model 3, further adjusted for HTN, T2DM, HF, AF, Cr, hs-CRP, and anxiolytic use. Restricted cubic spline models were used to separately evaluate potential nonlinear associations between CHG and HRV_z, between CHG and 1-year cardiovascular rehospitalization risk, and between HRV_z and 1-year cardiovascular rehospitalization risk. All analyses were performed using RStudio (R version 4.4.2), and a two-sided P < 0.05 was considered statistically significant. Odds ratios (ORs), regression coefficients (β), and 95% confidence intervals (CIs) were reported.

## Results

### Baseline characteristics

A total of 1020 patients were included in this study and categorized into four groups according to quartiles of the CHG index, with 255 patients in each quartile. Selected baseline characteristics are summarized in **Table 1**, and detailed baseline variables are provided in **Supplementary Table 1**. Overall, 55% of the participants were female, and the median age was 69 years. Age, sex, BMI, type 2 diabetes mellitus, cerebral infarction, chronic kidney disease, heart failure, atrial fibrillation, and anxiolytic use were generally comparable across CHG quartiles. Hypertension and hyperlipidemia differed significantly across groups. Patients in higher CHG quartiles showed higher fasting blood glucose, HbA1c, total cholesterol, triglycerides, and LDL-C, and lower HDL-C. hs-CRP levels also differed significantly across quartiles. The rehospitalization rates were similar in Q1 and Q2 but were higher in Q3 and Q4, with the highest rate observed in Q4.

**Table 1:** Selected baseline characteristics of the study population according to quartiles of the CHG index.

Characteristic	Overall	Q1	Q2	Q3	Q4	P-value
	N = 1020	N = 255	N = 255	N = 255	N = 255	
Sex						0.144
Female	566 (55%)	150 (59%)	127 (50%)	140 (55%)	149 (58%)	
Male	454 (45%)	105 (41%)	128 (50%)	115 (45%)	106 (42%)	
Age, years	69 (60, 75)	70 (61, 76)	68 (61, 74)	67 (59, 75)	68 (60, 75)	0.367
BMI, kg/m^2^	24.6 (22.4, 26.9)	24.4 (22.5, 27.0)	24.7 (22.7, 27.3)	24.2 (22.2, 26.7)	24.6 (22.2, 26.6)	0.522
HTN (%)						0.013
NO	218 (21%)	50 (20%)	43 (17%)	72 (28%)	53 (21%)	
YES	802 (79%)	205 (80%)	212 (83%)	183 (72%)	202 (79%)	
T2DM (%)						0.385
NO	678 (66%)	179 (70%)	162 (64%)	165 (65%)	172 (67%)	
YES	342 (34%)	76 (30%)	93 (36%)	90 (35%)	83 (33%)	
CI (%)						0.686
NO	691 (68%)	174 (68%)	176 (69%)	176 (69%)	165 (65%)	
YES	329 (32%)	81 (32%)	79 (31%)	79 (31%)	90 (35%)	
HLP (%)						<0.001
NO	785 (77%)	186 (73%)	180 (71%)	203 (80%)	216 (85%)	
YES	235 (23%)	69 (27%)	75 (29%)	52 (20%)	39 (15%)	
CKD (%)						0.698
NO	970 (95%)	241 (95%)	241 (95%)	246 (96%)	242 (95%)	
YES	50 (4.9%)	14 (5.5%)	14 (5.5%)	9 (3.5%)	13 (5.1%)	
HF (%)						0.279
NO	936 (92%)	240 (94%)	229 (90%)	236 (93%)	231 (91%)	
YES	84 (8.2%)	15 (5.9%)	26 (10%)	19 (7.5%)	24 (9.4%)	
AF (%)						0.399
NO	949 (93%)	235 (92%)	237 (93%)	243 (95%)	234 (92%)	
YES	71 (7.0%)	20 (7.8%)	18 (7.1%)	12 (4.7%)	21 (8.2%)	
Anxiolytic Use (%)						0.692
NO	582 (57%)	143 (56%)	151 (59%)	139 (55%)	149 (58%)	
YES	438 (43%)	112 (44%)	104 (41%)	116 (45%)	106 (42%)	
hs-CRP, mg/L	0.50 (0.50, 1.25)	0.50 (0.50, 0.63)	0.50 (0.50, 0.91)	0.50 (0.50, 1.67)	0.63 (0.50, 2.21)	<0.001
Cr, μmol/L	69 (59, 82)	71 (61, 82)	68 (57, 82)	69 (58, 81)	69 (58, 87)	0.483
FBG, mmol/L	5.10 (4.55, 6.00)	4.60 (4.23, 4.97)	4.89 (4.49, 5.38)	5.27 (4.69, 6.05)	6.57 (5.56, 8.10)	<0.001
HbA1c	6.10 (5.80, 6.70)	6.00 (5.70, 6.30)	6.00 (5.70, 6.30)	6.20 (5.80, 6.60)	6.70 (6.00, 7.70)	<0.001
TC, mmol/L	3.79 (3.09, 4.57)	3.18 (2.61, 3.64)	3.59 (3.02, 4.23)	4.06 (3.44, 4.88)	4.45 (3.72, 5.14)	<0.001
TG, mmol/L	1.25 (0.89, 1.76)	0.86 (0.66, 1.08)	1.16 (0.83, 1.48)	1.42 (1.10, 1.85)	1.78 (1.27, 2.64)	<0.001
HDL-C, mmol/L	1.17 (0.98, 1.40)	1.35 (1.10, 1.62)	1.19 (1.03, 1.41)	1.14 (0.98, 1.30)	1.01 (0.89, 1.21)	<0.001
LDL-C, mmol/L	1.99 (1.51, 2.60)	1.45 (1.15, 1.78)	1.90 (1.52, 2.27)	2.24 (1.81, 2.80)	2.59 (2.03, 3.01)	<0.001
1-year cardiovascular rehospitalization (%)						<0.001
NO	718 (70%)	206 (81%)	208 (82%)	171 (67%)	133 (52%)	
YES	302 (30%)	49 (19%)	47 (18%)	84 (33%)	122 (48%)	

Data are presented as n (%) or median (IQR). Group differences were compared using the chi-square test, Fisher’s exact test, or Kruskal–Wallis test, as appropriate. The quartile ranges of the CHG index were Q1: 2.94–4.77, Q2: >4.77–5.03, Q3: >5.03–5.31, and Q4: >5.31–6.96. Detailed baseline variables are shown in **Supplementary Table 1**. CHG, cholesterol–high-density lipoprotein–glucose; BMI, body mass index; HTN, hypertension; T2DM, type 2 diabetes mellitus; CI, cerebral infarction; HLP, hyperlipidemia; CKD, chronic kidney disease; HF, heart failure; AF, atrial fibrillation; hs-CRP, high-sensitivity C-reactive protein; Cr, creatinine; FBG, fasting blood glucose; HbA1c, glycated hemoglobin; TC, total cholesterol; TG, triglycerides; HDL-C, high-density lipoprotein cholesterol; LDL-C, low-density lipoprotein cholesterol.

### Baseline characteristics according to the availability of Holter-derived HRV data

To assess potential selection bias related to Holter data availability, we compared baseline characteristics between the Holter-available and Holter-unavailable groups. Overall, sex, age, BMI, most comorbidities, anxiolytic use, and the incidence of 1-year cardiovascular rehospitalization were generally comparable between the two groups. The 1-year cardiovascular rehospitalization rates were 30% and 29%, respectively (P = 0.685; SMD = 0.026). However, patients in the Holter-available group had a lower prevalence of heart failure and showed relatively lower levels of FBG, HbA1c, TC, TG, LDL-C, and CHG index, as well as higher HDL-C levels. The between-group difference was most pronounced for the CHG index (SMD = 0.336), and the absolute SMDs for TG, FBG, and LDL-C also exceeded 0.20 (**Supplementary Table 2**).

### Exploratory association between CHG and HRV_z in the Holter subgroup

In the Holter subgroup with complete 24-h ambulatory electrocardiography data, linear regression analyses showed an inverse association between the CHG index and HRV_z. When CHG was analyzed as a continuous variable, each 1-unit increase in CHG was associated with a 0.517-unit decrease in HRV_z in Model 1 (β = -0.517, 95% CI -0.673 to -0.361; P < 0.001). This association remained significant after adjustment for age, sex, and BMI (β = -0.509, 95% CI -0.666 to -0.352; P < 0.001), and was further maintained after additional adjustment for hypertension, type 2 diabetes mellitus, heart failure, atrial fibrillation, Cr, hs-CRP, and anxiolytic use (β = -0.492, 95% CI -0.648 to -0.336; P < 0.001). When CHG was analyzed by quartiles, compared with Q1, HRV_z did not differ significantly in Q2 or Q3, whereas only the highest quartile, Q4, showed a significant reduction in HRV_z, with consistent findings across all three models. Trend analyses showed an ordered decrease in HRV_z across increasing CHG quartiles (all P for trend < 0.001). However, given that Q2 and Q3 did not differ significantly from Q1, while Q4 showed a marked difference, these findings may suggest a nonlinear or threshold-like association between CHG and HRV_z rather than a simple linear dose-response decline (**Table 2**).

**Table 2:** Exploratory associations between the CHG index and HRV_z in multivariable linear regression analyses.

Variables	Model 1		Model 2		Model 3	
	β (95% CI)	P value	β(95% CI)	P value	β(95% CI)	P value
CHG continuous	-0.517 (-0.673, -0.361)	<0.001	-0.509 (-0.666, -0.352)	<0.001	-0.492 (-0.648, -0.336)	<0.001
CHG group	P for trend:<0.001		P for trend:<0.001		P for trend:<0.001	
Q1	Ref.		Ref.		Ref.	
Q2	-0.076 (-0.246, 0.093)	0.378	-0.082 (-0.252, 0.088)	0.345	-0.066 (-0.234, 0.102)	0.440
Q3	-0.114 (-0.284, 0.056)	0.188	-0.109 (-0.279, 0.061)	0.207	-0.095 (-0.262, 0.073)	0.268
Q4	-0.551 (-0.720, -0.381)	<0.001	-0.549 (-0.719, -0.379)	<0.001	-0.529 (-0.698, -0.361)	<0.001

Model 1: crude model.

Model 2: adjusted for age, sex, and BMI.

Model 3: further adjusted for hypertension, type 2 diabetes mellitus, atrial fibrillation, heart failure, Cr, hs-CRP, and anxiolytic use.

### Association between CHG and 1-year cardiovascular rehospitalization

Logistic regression analyses demonstrated that higher CHG was significantly associated with an increased risk of 1-year cardiovascular rehospitalization. When CHG was entered as a continuous variable, it remained significantly associated with 1-year cardiovascular rehospitalization across Models 1–3. In the fully adjusted model, each 1-unit increase in CHG was associated with a higher risk of rehospitalization (OR = 3.253, 95% CI 2.292–4.617; P < 0.001). When analyzed by quartiles, no significant difference was observed between Q2 and Q1, whereas Q3 and Q4 were associated with significantly increased risks of rehospitalization. In the fully adjusted model, the ORs were 1.957 (95% CI 1.296–2.955; P = 0.001) for Q3 and 3.656 (95% CI 2.443–5.472; P < 0.001) for Q4, compared with Q1. Trend analyses indicated a significant increase in the risk of 1-year cardiovascular rehospitalization across increasing CHG quartiles (all P for trend < 0.001) (**Table 3**).

**Table 3:** Associations between the CHG index and 1-year cardiovascular rehospitalization in logistic regression analyses.

Variables	Model 1		Model 2		Model 3	
	OR (95% CI)	P value	OR (95% CI)	P value	OR (95% CI)	P value
CHG continuous	3.361 (2.385, 4.736)	<0.001	3.425 (2.425, 4.837)	<0.001	3.253 (2.292, 4.617)	<0.001
CHG group	P for trend:<0.001		P for trend:<0.001		P for trend:<0.001	
Q1	Ref.		Ref.		Ref.	
Q2	0.967 (0.622, 1.505)	0.883	0.965 (0.620, 1.503)	0.875	0.907 (0.578, 1.424)	0.673
Q3	2.044 (1.364, 3.064)	<0.001	2.053 (1.368, 3.081)	<0.001	1.957 (1.296, 2.955)	0.001
Q4	3.827 (2.577, 5.682)	<0.001	3.866 (2.600, 5.747)	<0.001	3.656 (2.443, 5.472)	<0.001

Model 1: crude model.

Model 2: adjusted for age, sex, and BMI.

Model 3: further adjusted for hypertension, type 2 diabetes mellitus, atrial fibrillation, heart failure, Cr, hs-CRP, and anxiolytic use.

### Exploratory association between HRV_z and 1-year cardiovascular rehospitalization in the Holter subgroup

In the Holter subgroup, logistic regression analyses showed that higher HRV_z was associated with a lower risk of 1-year cardiovascular rehospitalization in the unadjusted model (OR = 0.716, 95% CI 0.562–0.912; P = 0.007). This association remained statistically significant after adjustment for age, sex, and BMI (OR = 0.715, 95% CI 0.561–0.912; P = 0.007). After full adjustment, HRV_z remained inversely associated with the risk of 1-year cardiovascular rehospitalization (OR = 0.706, 95% CI 0.550–0.907; P = 0.007) (**Table 4**).

**Table 4:** Exploratory association between HRV_z and 1-year cardiovascular rehospitalization in logistic regression analyses.

Model	Variable	OR (95% CI)	P value
Model 1	HRV_z	0.716 (0.562, 0.912)	0.007
Model 2	HRV_z	0.715 (0.561, 0.912)	0.007
Model 3	HRV_z	0.706 (0.550, 0.907)	0.007

Model 1: crude model.

Model 2: adjusted for age, sex, and BMI.

Model 3: further adjusted for hypertension, type 2 diabetes mellitus, atrial fibrillation, heart failure, Cr, hs-CRP, and anxiolytic use.

### Distribution of cardiovascular rehospitalization subtypes and exploratory subtype analyses

During the 1-year follow-up period, cardiovascular rehospitalization events were predominantly due to non-ACS ischemic chest pain or suspected myocardial ischemia, which occurred in 219 patients, accounting for 21.5% of the overall cohort and 72.5% of patients with cardiovascular rehospitalization. Other subtypes included arrhythmia-related rehospitalization in 29 patients, other cardiovascular causes in 26 patients, unplanned revascularization in 20 patients, worsening heart failure in 7 patients, and acute coronary syndrome in 1 patient.

Given the small number of events in the other subtypes, exploratory analyses were further performed for the predominant subtype. Higher CHG index was associated with an increased risk of rehospitalization for non-ACS ischemic chest pain or suspected myocardial ischemia. After multivariable adjustment, the OR for continuous CHG was 2.854 (95% CI 1.919-4.283; P < 0.001). In the Holter subgroup, higher HRV_z was associated with a lower risk of this subtype, with a multivariable-adjusted OR of 0.612 (95% CI 0.460-0.814; P < 0.001) (**Supplementary Table 3–5**).

### Restricted cubic spline analyses

**Figure 2:**
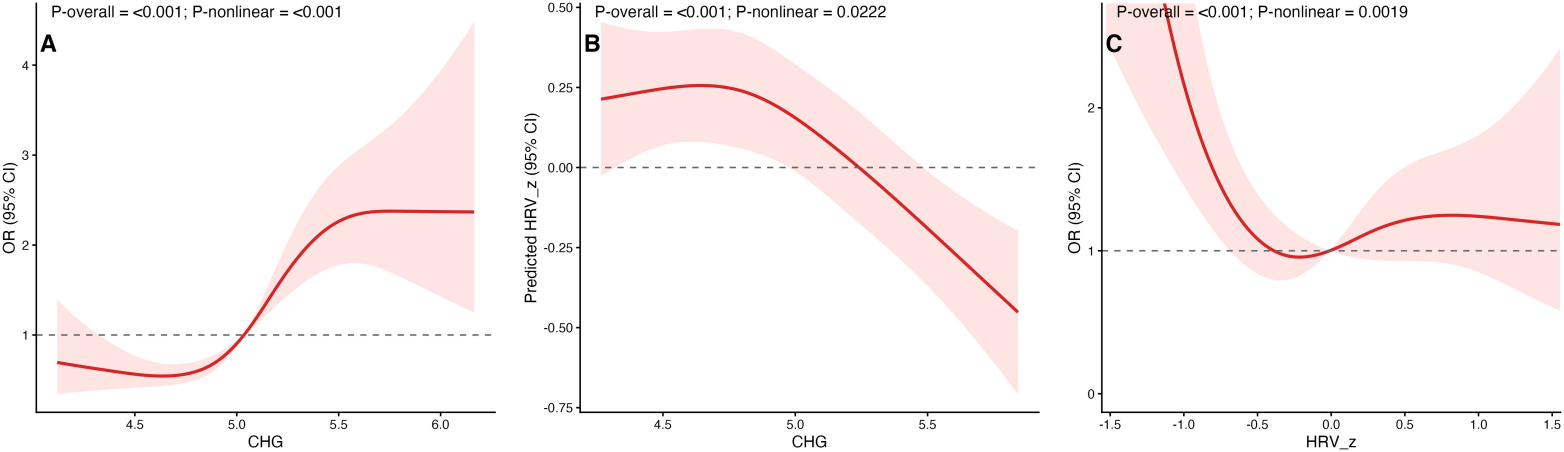
Restricted cubic spline analyses of the associations among the CHG index, HRV_z, and 1-year cardiovascular rehospitalization. **(A)** Association between the CHG index and 1-year cardiovascular rehospitalization. **(B)** Association between the CHG index and HRV_z. **(C)** Association between HRV_z and 1-year cardiovascular rehospitalization. Solid lines indicate estimated associations, and shaded areas indicate 95% confidence intervals. Models were adjusted for age, sex, BMI, hypertension, type 2 diabetes mellitus, heart failure, atrial fibrillation, creatinine, high-sensitivity C-reactive protein, and anxiolytic use.

Restricted cubic spline analyses revealed a nonlinear positive association between CHG and 1-year cardiovascular rehospitalization risk (P-overall < 0.001, P-nonlinear < 0.001). The risk of rehospitalization increased progressively with higher CHG levels and tended to plateau at higher levels. A nonlinear negative association was also observed between CHG and HRV_z (P-overall < 0.001, P-nonlinear = 0.0222), indicating that HRV_z tended to decrease more prominently at higher CHG levels. In addition, HRV_z showed a nonlinear negative association with 1-year cardiovascular rehospitalization risk (P-overall < 0.001, P-nonlinear = 0.0019). Patients with lower HRV_z had a higher rehospitalization risk, and the risk declined with increasing HRV_z before showing a slight upward trend at higher levels (**Figure 2**).

### Missing data and complete-case sensitivity analyses

In the final primary analytic cohort, the CHG index, CHG components, and 1-year cardiovascular rehospitalization outcome had no missing values. Missing data were mainly limited to BMI and hs-CRP, which were included as covariates in the analytic models. In the overall cohort, BMI was missing in 35 patients (3.4%) and hs-CRP in 40 patients (3.9%); 945 patients (92.6%) had complete data for both BMI and hs-CRP. In the Holter subgroup, HRV-related parameters and HRV_z had no missing values; BMI was missing in 20 patients (3.5%) and hs-CRP in 19 patients (3.3%); 532 patients (93.2%) had complete data for both BMI and hs-CRP. No patient in either cohort had both BMI and hs-CRP missing (**Supplementary Tables 6–7**).

Complete-case analyses were performed as sensitivity analyses and compared side by side with the main multiple-imputation analyses. Overall, the complete-case analyses did not materially change the direction of the main associations. However, because the HRV-related analyses were restricted to the Holter subgroup, these findings should still be interpreted cautiously in light of potential sample selection. In the Holter subgroup, the CHG index remained significantly and inversely associated with HRV_z in the fully adjusted model (β = -0.487, 95% CI -0.651 to -0.324; P < 0.001). In the overall cohort, the CHG index remained significantly associated with a higher risk of 1-year cardiovascular rehospitalization (OR = 3.648, 95% CI 2.515-5.292; P < 0.001). In addition, in the Holter subgroup, higher HRV_z remained associated with a lower risk of 1-year cardiovascular rehospitalization (OR = 0.650, 95% CI 0.499-0.845; P = 0.001) (**Supplementary Table 8**).

## Discussion

In this study of patients with CCS and comorbid anxiety, we evaluated the associations among the CHG index, HRV_z, and 1-year cardiovascular rehospitalization risk. Given the retrospective observational design, these findings should be interpreted as statistical associations rather than evidence of causality. The main findings were as follows. First, a higher CHG index was associated with lower HRV_z in the Holter subgroup. Second, a higher CHG index was associated with an increased risk of 1-year cardiovascular rehospitalization in the overall cohort. Third, lower HRV_z was associated with a higher risk of rehospitalization in exploratory analyses of the Holter subgroup. Fourth, restricted cubic spline analyses suggested nonlinear associations between CHG and HRV_z, between CHG and rehospitalization risk, and between HRV_z and rehospitalization risk. Collectively, these findings suggest that metabolic abnormalities, autonomic functional status, and adverse prognosis may be interrelated in patients with CCS and comorbid anxiety.

The CHG index is a novel composite marker integrating total cholesterol, fasting blood glucose, and high-density lipoprotein cholesterol, and may provide a broader reflection of glucolipid metabolic burden than individual metabolic indicators alone (19). In recent years, the CHG index has been increasingly used in the risk assessment of diabetes, cardiovascular disease, hypertension, and stroke (7, 9, 14, 15, 20–26). In the present study, a higher CHG index was associated with lower HRV_z, suggesting a possible relationship between glucolipid metabolic burden and autonomic regulation. Several mechanisms reported in previous studies may help explain this association. Hyperglycemia and insulin resistance can promote chronic low-grade inflammation, oxidative stress, and endothelial dysfunction, whereas dyslipidemia may further aggravate vascular reactivity abnormalities and neurohumoral dysregulation (27–30). These alterations could be related to sympathetic overactivation, reduced vagal tone, and lower HRV (31). As an established noninvasive marker of autonomic function, HRV reflects sympathovagal balance and the capacity to maintain physiological homeostasis (27, 32, 33). Therefore, the inverse association between CHG and HRV_z observed in this study may reflect a potential relationship between increasing metabolic burden and impaired autonomic regulation in this specific population.

We also found that HRV_z was inversely associated with 1-year cardiovascular rehospitalization risk in the Holter subgroup, suggesting that autonomic functional status may be related to prognosis in patients with CCS and comorbid anxiety. Reduced HRV generally reflects increased sympathetic activity and reduced vagal modulation. Such autonomic imbalance has been reported to be associated with impaired coronary blood flow and microcirculatory regulation, increased heart rate and blood pressure variability, and worsening myocardial oxygen supply-demand mismatch (10, 34, 35). In parallel, reduced vagal anti-inflammatory activity may be related to persistent inflammation and plaque instability, which may increase vulnerability to adverse cardiovascular events. In patients with coronary artery disease, impaired autonomic buffering capacity may also be associated with myocardial ischemia, arrhythmias, and recurrent symptoms, which could be associated with rehospitalization (36–39). Thus, HRV_z may serve as a physiological marker of autonomic status and may reflect neurocardiovascular vulnerability in patients with CCS and comorbid anxiety.

In addition to its association with HRV_z, an elevated CHG index may also be related to adverse clinical outcomes through metabolic and vascular mechanisms. In this study, a higher CHG index was associated with an increased risk of 1-year cardiovascular rehospitalization, suggesting that the metabolic burden captured by CHG may have clinical relevance beyond baseline risk characterization. Hyperglycemia and hypercholesterolemia can induce endothelial metabolic stress, promote inflammation, oxidative stress, and prothrombotic responses, and impair vasodilation and coronary microvascular regulation, thereby increasing susceptibility to recurrent ischemia and symptom relapse (15). In addition, elevated cholesterol promotes lipid deposition, foam cell formation, and plaque progression, whereas reduced HDL-C indicates impaired reverse cholesterol transport and weakened anti-inflammatory and antioxidant protection (30, 40). These mechanisms may partly explain the observed association between higher CHG and increased rehospitalization risk, although causal relationships cannot be inferred from the present observational data.

The present study focused specifically on patients with CCS and comorbid anxiety, a population in whom the combined assessment of metabolic status and autonomic function may be particularly relevant. Anxiety can activate the hypothalamic-pituitary-adrenal axis and sympathetic nervous system while suppressing vagal activity. It may also worsen cardiovascular risk through inflammation, sleep disturbance, reduced physical activity, and poorer treatment adherence (41–47). In this context, the metabolic burden reflected by a higher CHG index may coexist or interact with anxiety-related stress responses, autonomic imbalance, and behavioral risk factors, and may be related to a higher risk of rehospitalization. These findings support the potential value of integrated assessment of metabolic status, psychological status, and autonomic function in patients with CCS and comorbid anxiety.

In addition, restricted cubic spline analyses suggested nonlinear associations involving CHG, HRV_z, and rehospitalization risk, indicating that the risk patterns associated with these indicators may not be purely linear. For CHG, the associations with autonomic function and clinical outcomes appeared more pronounced at higher levels, suggesting a possible threshold-like pattern. Similarly, lower HRV_z was associated with a higher rehospitalization risk, whereas the risk pattern at higher HRV_z levels should be interpreted cautiously. These nonlinear findings suggest that, in patients with CCS and comorbid anxiety, the associations involving CHG and HRV_z may not reflect a simple linear accumulation of risk. CHG and HRV_z may help identify patients at higher risk, but the specific thresholds and their clinical implications require further validation in prospective studies.

Several limitations should be acknowledged. First, this was a single-center retrospective study and was therefore subject to selection bias and limited generalizability. Because of the observational design, unmeasured confounding cannot be excluded, and the findings should be interpreted as statistical associations rather than causal relationships. Second, anxiety was identified based on ICD-11 records during hospitalization, without standardized anxiety scale scores such as GAD-7 or HAMA. Therefore, anxiety severity, disease duration, and related clinical characteristics could not be further evaluated. In addition, only anxiolytic use was adjusted for, whereas medication categories, doses, treatment duration, and adherence were not available, leaving the possibility of residual confounding. Third, HRV-related analyses were restricted to patients with available Holter data. Although baseline characteristics were compared between patients with and without Holter data, Holter monitoring was not randomly performed and may have been influenced by clinical symptoms, disease severity, and physician judgment. Therefore, the associations involving HRV_z should be regarded as exploratory findings. Fourth, although multiple imputation was used and complete-case sensitivity analyses were performed, the missing-at-random assumption cannot be fully verified. In addition, this study captured only cardiovascular rehospitalizations occurring at the study center, and events at other hospitals or out-of-hospital deaths were not systematically available, which may have led to underestimation of outcome events. No independent endpoint adjudication committee was established, and subtype analyses were limited by the small number of events in several categories. Finally, no formal mediation analysis was performed. Therefore, the associations among CHG, HRV_z, and rehospitalization risk cannot confirm a causal “CHG–HRV_z–rehospitalization” pathway. Future multicenter prospective studies incorporating standardized psychological assessments, serial Holter-derived HRV monitoring, and more complete follow-up data are needed to validate these findings.

## Conclusions

In summary, in this single-center retrospective cohort, elevated CHG was associated with an increased risk of 1-year cardiovascular rehospitalization. In exploratory analyses of the Holter subgroup, higher CHG was associated with lower HRV_z, and lower HRV_z was also associated with a higher risk of rehospitalization. These findings suggest that metabolic abnormalities, autonomic functional status, and adverse prognosis may be interrelated in this population. As a simple integrated metabolic marker, CHG, together with Holter-derived HRV_z, may provide complementary information for risk assessment in patients with CCS and comorbid anxiety.

## Data Availability

The datasets generated and/or analyzed during the current study are not publicly available due to privacy and institutional restrictions but are available from the corresponding author on reasonable request and with permission from the relevant institution.
