# Supplementary material for: Cholesterol, high-density lipoprotein, and glucose index as a cardiometabolic marker associated with heart rate variability and 1-year cardiovascular rehospitalization in chronic coronary syndromes with comorbid anxiety: a retrospective cohort study
Source: Front Endocrinol (Lausanne). 2026 Jun 18;17:1871491. doi: 10.3389/fendo.2026.1871491 (PMC13322893; doi:10.3389/fendo.2026.1871491)
Supplement: Supplementary file 1 [file DataSheet1.docx]

**Supplementary Table S1. Detailed baseline characteristics of the study population according to quartiles of the CHG index.**

| Characteristic | Overall | Q1 | Q2 | Q3 | Q4 | p-value |
| --- | --- | --- | --- | --- | --- | --- |
|  | N = 1020 | N = 255 | N = 255 | N = 255 | N = 255 |  |
| Height,cm | 163 (158, 170) | 162 (158, 170) | 165 (158, 170) | 162 (157, 170) | 163 (157, 170) | 0.104 |
| Weight, kg | 65 (59, 74) | 65 (60, 74) | 68 (60, 75) | 65 (58, 73) | 65 (59, 72) | 0.142 |
| WBC,×10^9^/L | 5.96 (4.94, 7.12) | 5.39 (4.55, 6.74) | 5.83 (4.80, 6.83) | 6.20 (5.22, 7.42) | 6.33 (5.20, 7.49) | <0.001 |
| RBC,×10^12^/L | 4.34 (4.00, 4.70) | 4.23 (3.86, 4.58) | 4.22 (3.96, 4.60) | 4.38 (4.04, 4.70) | 4.51 (4.19, 4.87) | <0.001 |
| Hb,g/L | 132 (122, 143) | 128 (120, 139) | 131 (121, 140) | 133 (124, 143) | 137 (125, 148) | <0.001 |
| PLT,×10^9^/L | 197 (158, 235) | 186 (154, 227) | 203 (157, 241) | 204 (165, 233) | 196 (162, 238) | 0.039 |
| NEU,×10^9^/L | 3.67 (2.90, 4.66) | 3.34 (2.70, 4.16) | 3.61 (2.83, 4.50) | 3.74 (3.07, 4.74) | 4.06 (3.08, 5.05) | <0.001 |
| Lym,×10^9^/L | 1.60 (1.24, 2.01) | 1.46 (1.12, 1.83) | 1.59 (1.26, 1.96) | 1.70 (1.29, 2.14) | 1.62 (1.30, 2.08) | <0.001 |
| MO,×10^9^/L | 0.43 (0.34, 0.54) | 0.40 (0.32, 0.53) | 0.43 (0.35, 0.54) | 0.44 (0.33, 0.55) | 0.43 (0.36, 0.54) | 0.229 |
| ALT,U/L | 19 (16, 23) | 19 (16, 23) | 19 (16, 23) | 19 (16, 23) | 19 (16, 26) | 0.695 |
| AST,U/L | 18 (13, 26) | 17 (12, 23) | 17 (13, 24) | 18 (13, 26) | 19 (14, 31) | <0.001 |
| ALB,g/L | 40.0 (37.8, 42.5) | 39.1 (37.4, 41.2) | 39.8 (37.9, 41.9) | 40.4 (38.0, 43.1) | 41.0 (38.6, 43.7) | <0.001 |
| BUN,mmol/L | 5.50 (4.60, 6.64) | 5.44 (4.54, 6.41) | 5.38 (4.35, 6.52) | 5.50 (4.61, 6.73) | 5.69 (4.80, 7.03) | 0.033 |
| UA,μmol/L | 320 (263, 380) | 303 (242, 369) | 310 (257, 364) | 332 (275, 387) | 337 (280, 398) | <0.001 |
| ApoA1,g/L | 1.40 (1.25, 1.57) | 1.42 (1.33, 1.67) | 1.42 (1.18, 1.53) | 1.39 (1.26, 1.55) | 1.37 (1.24, 1.65) | 0.41 |
| ApoB,g/L | 0.73 (0.58, 0.92) | 0.55 (0.48, 0.64) | 0.66 (0.57, 0.80) | 0.82 (0.66, 0.94) | 0.94 (0.81, 1.09) | <0.001 |
| Lp(a),mg/L | 110 (55, 282) | 80 (48, 214) | 117 (61, 353) | 118 (54, 283) | 130 (52, 246) | 0.463 |
| hs-cTnI,pg/mL | 3.9 (2.6, 6.7) | 4.0 (2.6, 6.0) | 3.9 (2.6, 6.5) | 4.0 (2.7, 7.2) | 3.8 (2.7, 7.3) | 0.719 |
| NT-proBNP, pg/mL | 78 (39, 200) | 117 (59, 277) | 75 (40, 163) | 70 (32, 246) | 66 (34, 174) | 0.012 |
| PT,s | 13.10 (12.70, 13.60) | 13.20 (12.80, 13.70) | 13.20 (12.70, 13.60) | 13.00 (12.70, 13.50) | 12.90 (12.60, 13.40) | <0.001 |
| APTT,s | 35.0 (32.9, 37.5) | 35.7 (33.3, 37.8) | 35.2 (33.2, 37.9) | 35.0 (32.8, 37.5) | 34.5 (32.4, 37.1) | 0.037 |
| FIB,g/L | 2.96 (2.61, 3.39) | 2.85 (2.48, 3.25) | 2.97 (2.62, 3.36) | 2.97 (2.63, 3.42) | 3.10 (2.73, 3.60) | <0.001 |
| TT,s | 17.00 (16.40, 17.60) | 17.00 (16.30, 17.50) | 16.90 (16.40, 17.50) | 17.00 (16.40, 17.70) | 17.00 (16.50, 17.60) | 0.577 |
| D-Dimer,mg/L | 0.27 (0.22, 0.48) | 0.28 (0.22, 0.49) | 0.27 (0.22, 0.52) | 0.27 (0.22, 0.43) | 0.27 (0.22, 0.48) | 0.97 |

Data are presented as median (IQR). P values were calculated using the Kruskal–Wallis test. CHG quartiles were defined as follows: Q1, 2.94–4.77; Q2, 4.77–5.03; Q3, 5.03–5.31; and Q4, 5.31–6.96. CHG, cholesterol, high-density lipoprotein, and glucose; IQR, interquartile range; WBC, white blood cell count; RBC, red blood cell count; Hb, hemoglobin; PLT, platelet count; NEU, neutrophil count; Lym, lymphocyte count; MO, monocyte count; ALT, alanine aminotransferase; AST, aspartate aminotransferase; ALB, albumin; BUN, blood urea nitrogen; UA, uric acid; ApoA1, apolipoprotein A1; ApoB, apolipoprotein B; Lp(a), lipoprotein(a); hs-cTnI, high-sensitivity cardiac troponin I; NT-proBNP, N-terminal pro-B-type natriuretic peptide; PT, prothrombin time; APTT, activated partial thromboplastin time; FIB, fibrinogen; TT, thrombin time.

**Supplementary Table S2. Baseline characteristics according to availability of 24-hour Holter-derived HRV data**

| Characteristic | Overall  N = 1020 | Holter-available group  N = 571 | Holter-unavailable group  N = 449 | P value | SMD |
| --- | --- | --- | --- | --- | --- |
| Sex |  |  |  | 0.245 | 0.073 |
| Female | 566 (55%) | 326 (57%) | 240 (53%) |  |  |
| Male | 454 (45%) | 245 (43%) | 209 (47%) |  |  |
| Age, years | 69 (60, 75) | 69 (61, 75) | 68 (59, 75) | 0.345 | 0.061 |
| BMI, kg/m² | 24.6 (22.4, 26.9) | 24.4 (22.4, 27.0) | 24.8 (22.3, 26.9) | 0.344 | 0.060 |
| HTN (%) |  |  |  | 0.122 | 0.097 |
| NO | 218 (21%) | 112 (20%) | 106 (24%) |  |  |
| YES | 802 (79%) | 459 (80%) | 343 (76%) |  |  |
| T2DM (%) |  |  |  | 0.952 | 0.004 |
| NO | 678 (66%) | 380 (67%) | 298 (66%) |  |  |
| YES | 342 (34%) | 191 (33%) | 151 (34%) |  |  |
| CI (%) |  |  |  | 0.981 | 0.001 |
| NO | 691 (68%) | 387 (68%) | 304 (68%) |  |  |
| YES | 329 (32%) | 184 (32%) | 145 (32%) |  |  |
| HLP (%) |  |  |  | 0.118 | 0.099 |
| NO | 785 (77%) | 429 (75%) | 356 (79%) |  |  |
| YES | 235 (23%) | 142 (25%) | 93 (21%) |  |  |
| CKD (%) |  |  |  | 0.080 | 0.109 |
| NO | 970 (95%) | 549 (96%) | 421 (94%) |  |  |
| YES | 50 (4.9%) | 22 (3.9%) | 28 (6.2%) |  |  |
| HF (%) |  |  |  | 0.006 | 0.172 |
| NO | 936 (92%) | 536 (94%) | 400 (89%) |  |  |
| YES | 84 (8.2%) | 35 (6.1%) | 49 (11%) |  |  |
| AF (%) |  |  |  | 0.853 | 0.012 |
| NO | 949 (93%) | 532 (93%) | 417 (93%) |  |  |
| YES | 71 (7.0%) | 39 (6.8%) | 32 (7.1%) |  |  |
| Anxiolytic use(%) |  |  |  | 0.320 | 0.063 |
| NO | 582 (57%) | 318 (56%) | 264 (59%) |  |  |
| YES | 438 (43%) | 253 (44%) | 185 (41%) |  |  |
| hs-CRP, mg/L | 0.50 (0.50, 1.25) | 0.50 (0.50, 1.22) | 0.50 (0.50, 1.27) | 0.148 | 0.117 |
| Cr, μmol/L | 69 (59, 82) | 68 (59, 82) | 70 (58, 83) | 0.601 | 0.093 |
| FBG, mmol/L | 5.10 (4.55, 6.00) | 5.01 (4.49, 5.70) | 5.23 (4.63, 6.27) | <0.001 | 0.215 |
| HbA1c | 6.10 (5.80, 6.70) | 6.10 (5.80, 6.60) | 6.20 (5.80, 6.80) | 0.016 | 0.180 |
| TC, mmol/L | 3.79 (3.09, 4.57) | 3.69 (3.04, 4.47) | 3.93 (3.14, 4.69) | 0.010 | 0.166 |
| TG, mmol/L | 1.25 (0.89, 1.76) | 1.18 (0.85, 1.66) | 1.34 (0.96, 1.88) | <0.001 | 0.258 |
| HDL-C, mmol/L | 1.17 (0.98, 1.40) | 1.18 (1.00, 1.43) | 1.13 (0.96, 1.35) | 0.011 | 0.128 |
| LDL-C, mmol/L | 1.99 (1.51, 2.60) | 1.89 (1.47, 2.51) | 2.13 (1.58, 2.68) | <0.001 | 0.213 |
| CHG index | 5.03 (4.77, 5.31) | 4.97 (4.71, 5.24) | 5.09 (4.83, 5.39) | <0.001 | 0.336 |
| WBC, ×10⁹/L | 5.96 (4.94, 7.12) | 5.89 (4.85, 7.15) | 6.08 (5.00, 7.09) | 0.193 | 0.111 |
| RBC, ×10¹²/L | 4.34 (4.00, 4.70) | 4.30 (3.99, 4.69) | 4.38 (4.00, 4.73) | 0.221 | 0.094 |
| Hb, g/L | 132 (122, 143) | 131 (122, 142) | 132 (123, 144) | 0.370 | 0.063 |
| PLT, ×10⁹/L | 197 (158, 235) | 196 (158, 235) | 197 (159, 235) | 0.726 | 0.039 |
| NEU, ×10⁹/L | 3.67 (2.90, 4.66) | 3.65 (2.90, 4.58) | 3.70 (2.89, 4.80) | 0.528 | 0.089 |
| Lym, ×10⁹/L | 1.60 (1.24, 2.01) | 1.58 (1.21, 2.00) | 1.64 (1.31, 2.03) | 0.060 | 0.027 |
| MO, ×10⁹/L | 0.43 (0.34, 0.54) | 0.43 (0.33, 0.54) | 0.43 (0.34, 0.54) | 0.919 | 0.051 |
| ALT, U/L | 19 (16, 23) | 19 (16, 23) | 19 (16, 24) | 0.622 | 0.012 |
| AST, U/L | 18 (13, 26) | 18 (13, 25) | 17 (13, 26) | 0.583 | 0.049 |
| ALB, g/L | 40.0 (37.8, 42.5) | 39.8 (37.5, 42.3) | 40.2 (38.3, 42.6) | 0.019 | 0.134 |
| BUN, mmol/L | 5.50 (4.60, 6.64) | 5.48 (4.53, 6.58) | 5.58 (4.67, 6.75) | 0.277 | 0.043 |
| UA, μmol/L | 320 (263, 380) | 317 (260, 379) | 322 (268, 387) | 0.274 | 0.077 |
| ApoA1, g/L | 1.40 (1.25, 1.57) | 1.42 (1.27, 1.62) | 1.37 (1.25, 1.55) | 0.502 | 0.022 |
| ApoB, g/L | 0.73 (0.58, 0.92) | 0.66 (0.55, 0.91) | 0.80 (0.60, 0.95) | 0.068 | 0.179 |
| Lp(a), mg/L | 110 (55, 282) | 118 (57, 258) | 92 (51, 283) | 0.406 | 0.104 |
| PT, s | 13.10 (12.70, 13.60) | 13.10 (12.70, 13.60) | 13.00 (12.60, 13.50) | 0.033 | 0.072 |
| APTT, s | 35.0 (32.9, 37.5) | 35.2 (33.1, 37.6) | 34.9 (32.7, 37.2) | 0.112 | 0.092 |
| FIB, g/L | 2.96 (2.61, 3.39) | 2.94 (2.59, 3.32) | 3.01 (2.62, 3.49) | 0.097 | 0.011 |
| TT, s | 17.00 (16.40, 17.60) | 17.00 (16.40, 17.60) | 17.00 (16.40, 17.60) | 0.981 | 0.076 |
| D-dimer, mg/L | 0.27 (0.22, 0.48) | 0.27 (0.22, 0.46) | 0.29 (0.22, 0.50) | 0.244 | 0.043 |
| 1-year cardiovascular rehospitalization | | |  | 0.685 | 0.026 |
| NO | 718 (70%) | 399 (70%) | 319 (71%) |  |  |
| YES | 302 (30%) | 172 (30%) | 130 (29%) |  |  |

Data are presented as median (interquartile range) for continuous variables and n (%) for categorical variables. P values were calculated using the Wilcoxon rank-sum test for continuous variables and the chi-square test or Fisher’s exact test for categorical variables, as appropriate. Standardized mean differences are presented as absolute values, with an absolute SMD <0.10 considered negligible. The Holter-available group included patients with complete 24-hour Holter-derived HRV parameters, including SDNN, SDANN, rMSSD, and pNN50. CHG, cholesterol–high-density lipoprotein–glucose index; HRV, heart rate variability; SMD, standardized mean difference.

# **Supplementary Table S3. Distribution of cardiovascular rehospitalization subtypes**

| subtype label | patients (n) | Patients, n (%) of total cohort | Patients, n (%) of rehospitalized patients |
| --- | --- | --- | --- |
| Acute coronary syndrome, including unstable angina and myocardial infarction | 1 | 1 (0.1%) | 1 (0.3%) |
| Non-ACS ischemic chest pain or suspected myocardial ischemia | 219 | 219 (21.5%) | 219 (72.5%) |
| Worsening heart failure | 7 | 7 (0.7%) | 7 (2.3%) |
| Arrhythmia-related rehospitalization | 29 | 29 (2.8%) | 29 (9.6%) |
| Unplanned revascularization | 20 | 20 (2.0%) | 20 (6.6%) |
| Other cardiovascular causes | 26 | 26 (2.5%) | 26 (8.6%) |

Values are presented as the number and percentage of unique patients. Percentages were calculated using the total cohort and patients with cardiovascular rehospitalization as denominators, respectively. ACS, acute coronary syndrome.Each patient was classified according to the primary reason for cardiovascular rehospitalization and was counted only once.

**Supplementary Table S4. Exploratory association of the CHG index with Non-ACS ischemic chest pain or suspected myocardial ischemia**

| Variables | Model 1 | | Model 2 | | Model 3 | |
| --- | --- | --- | --- | --- | --- | --- |
|  | OR (95% CI) | P value | OR (95% CI) | P value | OR (95% CI) | P value |
| CHG continuous | 2.761 (1.914, 4.016) | <0.001 | 3.002 (2.059, 4.418) | <0.001 | 2.854 (1.919, 4.283) | <0.001 |
| CHG group | P for trend：<0.001 | | P for trend：<0.001 | | P for trend：<0.001 | |
| Q1 | Ref. | | Ref. | | Ref. | |
| Q2 | 0.855 (0.536, 1.360) | 0.509 | 0.901 (0.553, 1.464) | 0.673 | 0.898 (0.541, 1.487) | 0.675 |
| Q3 | 1.388 (0.894, 2.163) | 0.145 | 1.562 (0.988, 2.484) | 0.057 | 1.670 (1.038, 2.707) | 0.036 |
| Q4 | 3.104 (2.059, 4.730) | <0.001 | 3.348 (2.184, 5.203) | <0.001 | 3.190 (2.033, 5.077) | <0.001 |

The number of outcome events was 219.Odds ratios (ORs) and 95% confidence intervals (CIs) were estimated using logistic regression. The outcome was Non-ACS ischemic chest pain or suspected myocardial ischemia. Patients without cardiovascular rehospitalization served as the reference group, and patients with other cardiovascular rehospitalization subtypes were excluded. Q1 was used as the reference category for CHG quartiles. P for trend was calculated by modeling CHG quartiles as an ordinal variable. Model 1 was unadjusted; Model 2 was adjusted for age, sex, and BMI; Model 3 was further adjusted for hypertension, type 2 diabetes mellitus, heart failure, atrial fibrillation, Cr, hs-CRP, and anxiolytic use.

**Supplementary Table S5. Exploratory association of HRV_z with Non-ACS ischemic chest pain or suspected myocardial ischemia in the Holter subgroup**

| Model | Variable | OR (95% CI) | P value |
| --- | --- | --- | --- |
| Model 1 | HRV_z | 0.644 (0.491, 0.845) | 0.002 |
| Model 2 | HRV_z | 0.630 (0.478, 0.831) | 0.001 |
| Model 3 | HRV_z | 0.612 (0.460, 0.814) | <0.001 |

Odds ratios (ORs) and 95% confidence intervals (CIs) were estimated using logistic regression. The outcome was non-ACS ischemic chest pain or suspected myocardial ischemia. This exploratory analysis was restricted to patients with available 24-hour Holter-derived HRV parameters. Among the 571 patients in the Holter subgroup, 531 were included in this subtype-specific analysis, including 132 patients with non-ACS ischemic chest pain or suspected myocardial ischemia and 399 patients without cardiovascular rehospitalization. Forty patients with other cardiovascular rehospitalization subtypes were excluded. HRV_z was analyzed as a continuous variable per 1-unit increase. Model 1 was unadjusted; Model 2 was adjusted for age, sex, and BMI; Model 3 was further adjusted for hypertension, type 2 diabetes mellitus, heart failure, atrial fibrillation, Cr, hs-CRP, and anxiolytic use. OR, odds ratio; CI, confidence interval; ACS, acute coronary syndrome; HRV, heart rate variability; Cr, creatinine; hs-CRP, high-sensitivity C-reactive protein.

**Supplementary Table S6. Extent of missing data and imputation methods**

| **Variable** | **Role in analysis** | **Observed, n** | **Missing, n (%)** | **Imputed?** | **Imputation method** |
| --- | --- | --- | --- | --- | --- |
| **Panel A. Main cohort（N = 1020）** | | | | | |
| Age | Covariate | 1,020 | 0 (0.0%) | No | Not imputed |
| Sex | Covariate | 1,020 | 0 (0.0%) | No | Not imputed |
| BMI | Covariate | 985 | 35 (3.4%) | Yes | Predictive mean matching |
| HTN | Covariate | 1,020 | 0 (0.0%) | No | Not imputed |
| T2DM | Covariate | 1,020 | 0 (0.0%) | No | Not imputed |
| HF | Covariate | 1,020 | 0 (0.0%) | No | Not imputed |
| AF | Covariate | 1,020 | 0 (0.0%) | No | Not imputed |
| Cr | Covariate | 1,020 | 0 (0.0%) | No | Not imputed |
| hs-CRP | Covariate | 980 | 40 (3.9%) | Yes | Predictive mean matching |
| Anxiolytic use | Covariate | 1,020 | 0 (0.0%) | No | Not imputed |
| TC | CHG component | 1,020 | 0 (0.0%) | No | Not imputed |
| HDL-C | CHG component | 1,020 | 0 (0.0%) | No | Not imputed |
| FBG | CHG component | 1,020 | 0 (0.0%) | No | Not imputed |
| CHG index | Exposure | 1,020 | 0 (0.0%) | No | Not imputed |
| 1-year cardiovascular rehospitalization | Outcome | 1,020 | 0 (0.0%) | No | Not imputed |
| **Panel B. Holter subgroup（N=571）** | | | | | |
| Age | Covariate | 571 | 0 (0.0%) | No | Not imputed |
| Sex | Covariate | 571 | 0 (0.0%) | No | Not imputed |
| BMI | Covariate | 551 | 20 (3.5%) | Yes | Predictive mean matching |
| HTN | Covariate | 571 | 0 (0.0%) | No | Not imputed |
| T2DM | Covariate | 571 | 0 (0.0%) | No | Not imputed |
| HF | Covariate | 571 | 0 (0.0%) | No | Not imputed |
| AF | Covariate | 571 | 0 (0.0%) | No | Not imputed |
| Cr | Covariate | 571 | 0 (0.0%) | No | Not imputed |
| hs-CRP | Covariate | 552 | 19 (3.3%) | Yes | Predictive mean matching |
| Anxiolytic use | Covariate | 571 | 0 (0.0%) | No | Not imputed |
| TC | CHG component | 571 | 0 (0.0%) | No | Not imputed |
| HDL-C | CHG component | 571 | 0 (0.0%) | No | Not imputed |
| FBG | CHG component | 571 | 0 (0.0%) | No | Not imputed |
| CHG index | Exposure | 571 | 0 (0.0%) | No | Not imputed |
| SDNN | HRV component | 571 | 0 (0.0%) | No | Not imputed |
| SDANN | HRV component | 571 | 0 (0.0%) | No | Not imputed |
| rMSSD | HRV component | 571 | 0 (0.0%) | No | Not imputed |
| pNN50 | HRV component | 571 | 0 (0.0%) | No | Not imputed |
| 1-year cardiovascular rehospitalization | Outcome | 571 | 0 (0.0%) | No | Not imputed |

Missingness was assessed after applying the eligibility criteria and before multiple imputation. Body mass index and high-sensitivity C-reactive protein were imputed using multiple imputation by chained equations with predictive mean matching. CHG components, the CHG index, Holter-derived HRV components, HRV_z, and 1-year cardiovascular rehospitalization outcome were not imputed. HRV variables were assessed only in the Holter subgroup.

**Supplementary Table S7. Missing-data patterns in the main cohort and Holter subgroup**

| Missing-data pattern | Body mass index | High-sensitivity C-reactive protein | Main cohort, n (%) N = 1020 | Holter subgroup, n (%) N = 571 |
| --- | --- | --- | --- | --- |
| Complete for BMI and hs-CRP | Observed | Observed | 945 (92.6%) | 532 (93.2%) |
| BMI missing only | Missing | Observed | 35 (3.4%) | 20 (3.5%) |
| hs-CRP missing only | Observed | Missing | 40 (3.9%) | 19 (3.3%) |
| Both BMI and hs-CRP missing | Missing | Missing | 0 (0.0%) | 0 (0.0%) |

Missing-data patterns were assessed after applying the study eligibility criteria and before multiple imputation. This table summarizes the joint missingness of variables with missing observations in the analysis models. Missingness was limited to body mass index and high-sensitivity C-reactive protein. CHG components, the CHG index, Holter-derived HRV components, HRV_z, and the 1-year cardiovascular rehospitalization outcome were not imputed.

**Supplementary Table S8. Comparison of primary multiple-imputation analyses with complete-case sensitivity analyses**

Panel A. Association between CHG index and HRV_z in the Holter subgroup

| Model | Analysis sample | Multiple-imputation analysis, N | Multiple-imputation estimate | Complete-case analysis, N | Complete-case estimate |
| --- | --- | --- | --- | --- | --- |
| Model 1 | Holter subgroup | 571 | β = -0.517 (-0.673, -0.361); P < 0.001 | 571 | β = -0.516 (-0.673, -0.359);  P < 0.001 |
| Model 2 | Holter subgroup | 571 | β = -0.509 (-0.666, -0.352);  P < 0.001 | 551 | β = -0.497 (-0.658, -0.336);  P < 0.001 |
| Model 3 | Holter subgroup | 571 | β = -0.492 (-0.648, -0.336);  P < 0.001 | 532 | β = -0.487 (-0.651, -0.324);  P < 0.001 |

Panel B. Association between CHG index and 1-year cardiovascular rehospitalization in the main cohort

| Model | Analysis sample | Multiple-imputation analysis, N/events | Multiple-imputation estimate | Complete-case analysis, N/events | Complete-case estimate |
| --- | --- | --- | --- | --- | --- |
| Model 1 | Main cohort | 1020 / 302 | OR = 3.361 (2.385, 4.736);  P < 0.001 | 1020 / 302 | OR = 3.412 (2.418, 4.815);  P < 0.001 |
| Model 2 | Main cohort | 1020 / 302 | OR = 3.425 (2.425, 4.837);  P < 0.001 | 985 / 289 | OR = 3.735 (2.617, 5.332);  P < 0.001 |
| Model 3 | Main cohort | 1020 / 302 | OR = 3.253 (2.292, 4.617);  P < 0.001 | 945 / 277 | OR = 3.648 (2.515, 5.292);  P < 0.001 |

Panel C. Association between HRV_z and 1-year cardiovascular rehospitalization in the Holter subgroup

| Model | Analysis sample | Multiple-imputation analysis, N/events | Multiple-imputation estimate | Complete-case analysis, N/events | Complete-case estimate |
| --- | --- | --- | --- | --- | --- |
| Model 1 | Holter subgroup | 571 / 172 | OR = 0.716 (0.562, 0.912);  P = 0.007 | 571 / 172 | OR = 0.716 (0.562, 0.910); P = 0.006 |
| Model 2 | Holter subgroup | 571 / 172 | OR = 0.715 (0.561, 0.912);  P = 0.007 | 551 / 162 | OR = 0.673 (0.525, 0.864); P = 0.002 |
| Model 3 | Holter subgroup | 571 / 172 | OR = 0.706 (0.550, 0.907);  P = 0.007 | 532 / 157 | OR = 0.650 (0.499, 0.845); P = 0.001 |

Multiple-imputation analyses were considered the primary analyses, whereas complete-case analyses were performed as sensitivity analyses. Estimates are presented per 1-unit increase in the CHG index or HRV_z. Model 1 was unadjusted. Model 2 was adjusted for age, sex, and body mass index. Model 3 was adjusted for age, sex, body mass index, hypertension, type 2 diabetes mellitus, heart failure, atrial fibrillation, creatinine, high-sensitivity C-reactive protein, and anxiolytic use. β values were estimated using linear regression for HRV_z, and odds ratios were estimated using logistic regression for 1-year cardiovascular rehospitalization.

**Supplementary Figure S1. Flow diagram of multiple-imputation and complete-case analyses in the overall cohort and Holter subgroup.**

Flow diagram of multiple-imputation and complete-case analyses in the overall cohort and Holter subgroup. The final main analysis cohort included 1020 patients with available CHG index and 1-year cardiovascular rehospitalization data. Multiple-imputation analyses were performed in the overall cohort and in the Holter subgroup, with BMI and hs-CRP imputed as baseline covariates. Complete-case analyses were conducted for the fully adjusted models using patients with complete BMI and hs-CRP data.


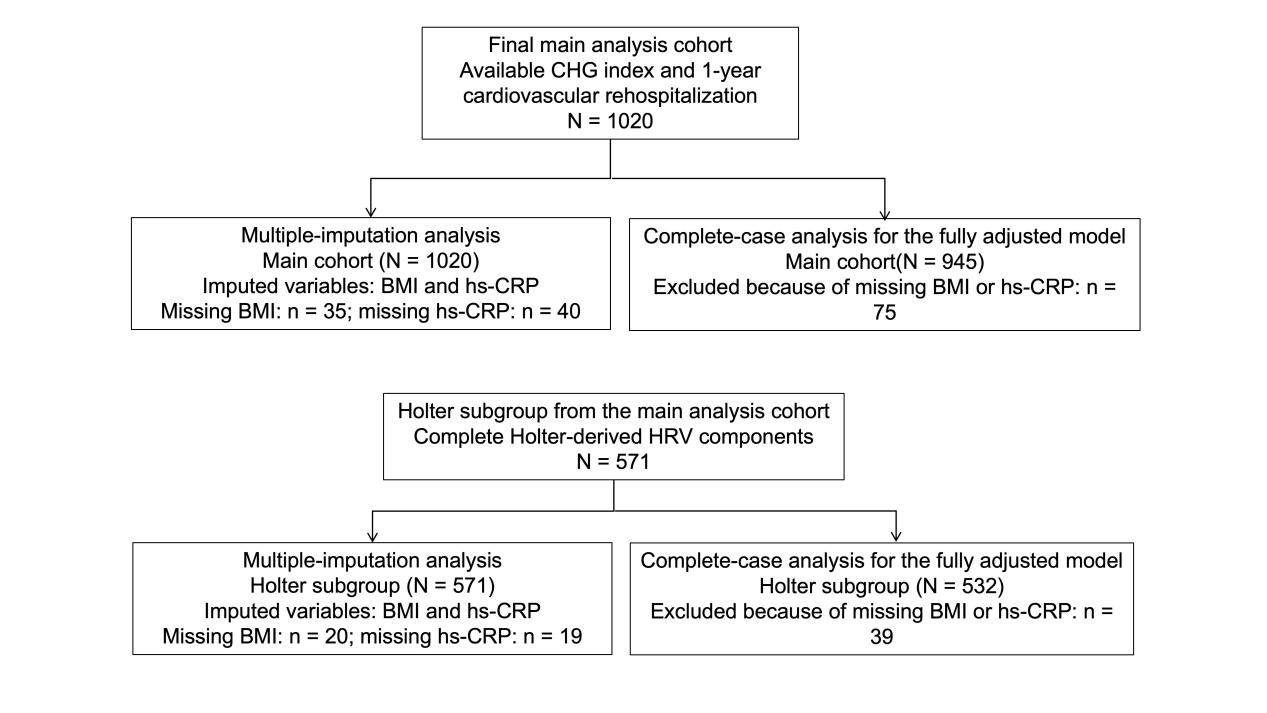


**Supplementary Figure S2. Representative multiple-imputation diagnostic plots for BMI and hs-CRP. Panels A and B show trace plots for the overall cohort and Holter subgroup, respectively. Panels C and D show density plots for imputed BMI values, and Panels E and F show density plots for imputed hs-CRP values.**


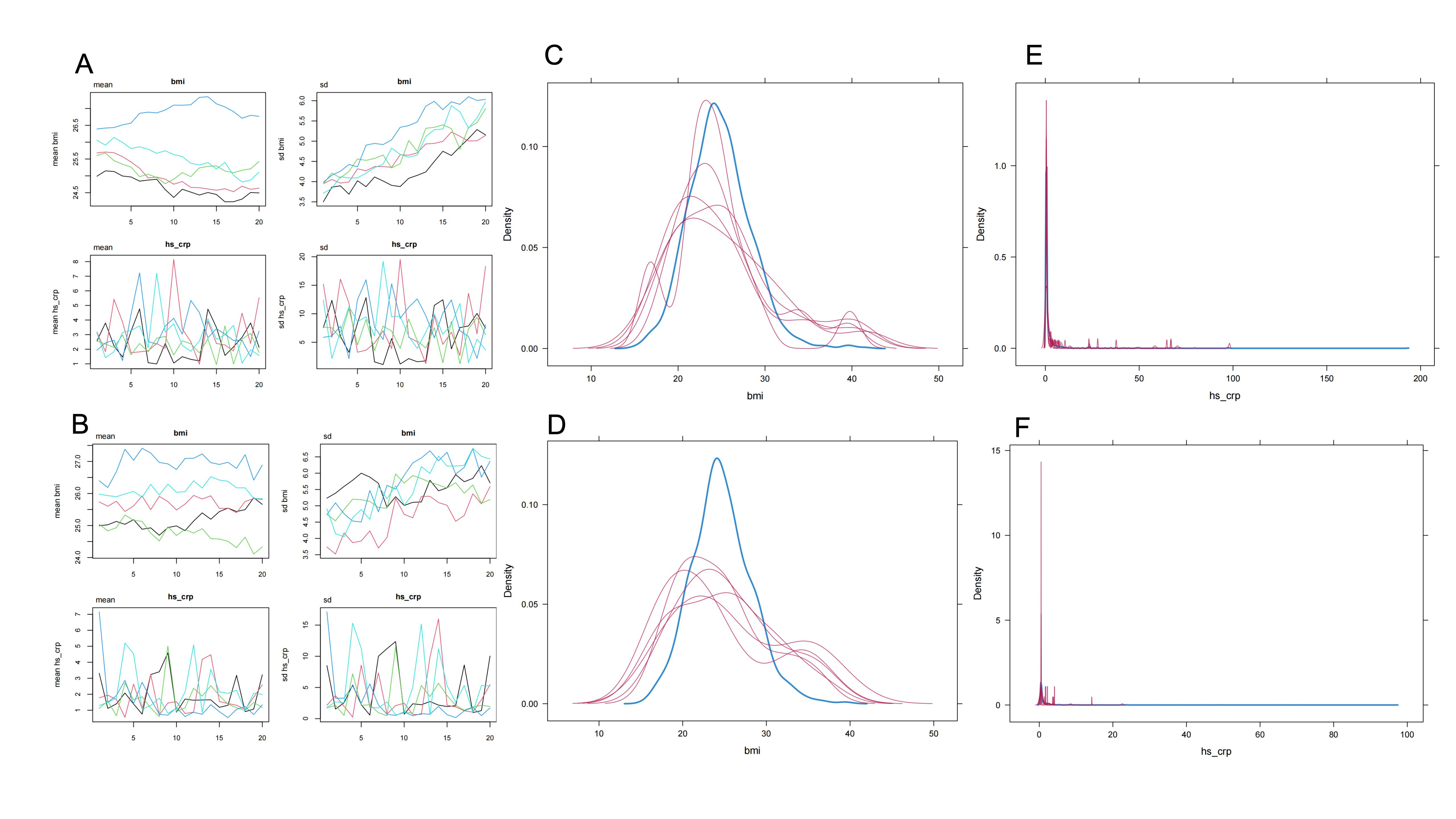


Representative diagnostic plots for multiple imputation of BMI and hs-CRP. Panels A and B show trace plots for the overall cohort and Holter subgroup, respectively. Panels C and D show density plots comparing observed and imputed BMI distributions in the overall cohort and Holter subgroup, respectively. Panels E and F show density plots comparing observed and imputed hs-CRP distributions in the overall cohort and Holter subgroup, respectively.
